# Hyperspectral estimation of leaf chlorophyll under small-sample conditions via spectral augmentation and weighted ensemble learning

**DOI:** 10.3389/fpls.2026.1851448

**Published:** 2026-05-20

**Authors:** Jiahui Xu, Yalong Song, Yuanyuan Liu, Shuo Zhang, Guansan Zhu, Wentao Li, Xufeng Wang, Jianfei Xing

**Affiliations:** 1 College of Mechanical and Electrical Engineering, Tarim University, Alar, China; 2 Xinjiang Production and Construction Corps Key Laboratory of Utilization and Equipment of Special Agricultural and Forestry Products in Southern Xinjiang, Alar, China; 3 Modern Agricultural Engineering Key Laboratory, Universities of Education Department of Xinjiang Uygur, Autonomous Region, Alar, China; 4 College of Engineering, China Agricultural University, Beijing, China

**Keywords:** chlorophyll, data augmentation, ensemble learning, Optuna, small-sample

## Abstract

**Introduction:**

Under small-sample conditions, hyperspectral leaf chlorophyll estimation is affected by high-dimensional collinearity, measurement noise, and cross-source acquisition discrepancies. Existing studies often treat training-distribution expansion and model-error complementarity separately. This study proposed a physically constrained composite spectral augmentation–weighted ensemble framework for reproducible small-sample chlorophyll estimation.

**Methods:**

Using 1,113 valid spectrum–label pairs from the leaf subset of the GreenHySpectra dataset in the 400–1000 nm range, spectra and chlorophyll reference values were matched by sample identifiers and divided into training and validation sets. Low-magnitude Gaussian noise and smooth wavelength warping were applied only to the training set. XGBoost, partial least squares regression, and ridge regression were optimized with Optuna using a CMA-ES sampler, and ensemble weights were calibrated by Bayesian optimization. An independent external set of 90 tomato leaf samples was used to evaluate transferability.

**Results:**

Composite augmentation improved model stability and reduced validation error relative to the non-augmented baseline. The weighted ensemble model achieved the best internal performance, with R² = 0.6392 and RMSE = 8.8883. On the external samples, the model achieved R² = 0.498 and RMSE = 9.801.

**Discussion:**

The proposed workflow integrates physically plausible augmentation, heterogeneous learner complementarity, and independent external validation. The external results indicate partial cross-source transferability while highlighting distributional and measurement-chain discrepancies that still limit absolute generalization.

## 1 Introduction

Chlorophyll is a core pigment in photosynthesis, and its temporal variation is closely linked to plant nitrogen status, photosynthetic capacity, and stress responses. Rapid and non-destructive estimation of chlorophyll content is therefore important for crop phenotyping and precision agriculture (Blackburn, 2007; Khan et al., 2024). Traditional chlorophyll determination relies mainly on destructive wet-chemistry protocols, typically based on solvent extraction and spectrophotometry. Although these methods are accurate, they are labor-intensive and unsuitable for large-scale or repeated monitoring (Lichtenthaler, 1987). Reflectance spectroscopy offers a non-destructive alternative because chlorophyll strongly affects the visible absorption region and the red-edge transition zone (Blackburn, 2007; Wang et al., 2018). Chlorophyll-related estimation has therefore been approached through empirical indices, red-edge features, and mechanistic radiative-transfer models such as PROSPECT and PROSAIL (Gitelson et al., 1996; Sims and Gamon, 2002; Curran, 1989; Jacquemoud and Baret, 1990; Verhoef, 1984).

To improve robustness under small-sample conditions, data augmentation can expand the effective training distribution through controlled perturbations (Shorten and Khoshgoftaar, 2019). Although most systematic work on augmentation began in computer vision, similar ideas have also been extended to hyperspectral data, including perturbation strategies that preserve spectral structure and improve model generalization (Nalepa et al., 2019). The perturbation strength, however, must remain physically plausible; excessive noise or excessive warping can distort meaningful absorption patterns and degrade model performance (Nalepa et al., 2019; Rinnan et al., 2009). Ensemble learning provides a complementary route to robustness by combining learners with different inductive biases, often reducing both variance and sensitivity to distribution shift (Dietterich, 2000; Zhou, 2012). In this context, nonlinear learners such as XGBoost can capture interaction effects, whereas regularized linear models such as ridge regression can provide stable predictions under strong collinearity (Chen and Guestrin, 2016; Hoerl and Kennard, 1970).

Although related research has made progress in areas such as spectral preprocessing, feature selection, single-model optimization, and deep learning, robust chlorophyll estimation remains challenging under the simultaneous presence of “small sample size, source heterogeneity, and high-dimensional collinearity (Jacquemoud et al., 2009; Verrelst et al., 2015).” Existing methods tend to address these issues separately: one line of work emphasizes improving model architecture while implicitly assuming that the training distribution sufficiently represents subsequent acquisition perturbations; the other focuses on augmentation or mechanistic constraints (Hoerl and Kennard, 1970), yet rarely examines whether these perturbations can maintain their benefits on independent external samples (Wold et al., 2001). Consequently, performance gains observed in internal validation do not necessarily transfer to cross-location, cross-batch, or cross-instrument samples (Chen and Guestrin, 2016). Therefore, the core challenge faced in this paper is not “whether an even more complex model can be introduced,” but rather how to simultaneously mitigate input distribution shift and single-model generalization instability under sample-limited conditions, in a reproducible and interpretable manner (Akiba et al., 2019; Hansen and Ostermeier, 2001; Snoek et al., 2012).

Based on the above considerations, this paper positions its research as a modeling framework that emphasizes reproducibility and the identification of transfer boundaries, rather than merely pursuing higher complexity (Shorten and Khoshgoftaar, 2019; Nalepa et al., 2019; Rinnan et al., 2009). The core contributions of this paper are as follows: (1) We propose a physically constrained composite spectral augmentation strategy that sequentially combines low-amplitude Gaussian noise with smooth spectral warping to simulate measurement noise and continuum spectral drift. (2) We construct a heterogeneous weighted ensemble consisting of PLS, Ridge, and XGBoost, achieving unified hyperparameter tuning and weight calibration using Optuna and Bayesian optimization. (3) Under the conditions of a fixed random seed, strict training–validation separation, and independent external sample testing, we discuss the effective domain of the proposed method and its external transferability, without equating internal performance improvement directly with universal generalization capability.

## 2 Materials and methods

### 2.1 Materials

#### 2.1.1 The GreenHySpectra dataset

This study used the GreenHySpectra dataset, a multi-source hyperspectral resource that contains plant reflectance measurements from multiple projects and environments (Cherif et al., 2025; Avatarr05, 2023). The full dataset spans 400–2450 nm with 1,721 bands. For the present chlorophyll regression task, only the leaf subset with matched chlorophyll-related labels was used.

After download, the public dataset was screened for valid spectrum–label pairs and missing-value patterns. Because chlorophyll-sensitive information is concentrated in the visible, red-edge, and near-infrared region, whereas the SWIR portion contained substantial gaps, we retained the 400–1000 nm range for modeling (Gao, 1996; Green et al., 1998). This subset preserves the main chlorophyll-sensitive spectral structure while avoiding heavily missing SWIR segments. Within this range, the spectra show the expected vegetation pattern, including strong visible absorption and a clear red-edge transition; the band near 970–980 nm also reflects leaf-water-related absorption (Sims and Gamon, 2002; Ceccato et al., 2002).

**Figure 1:**
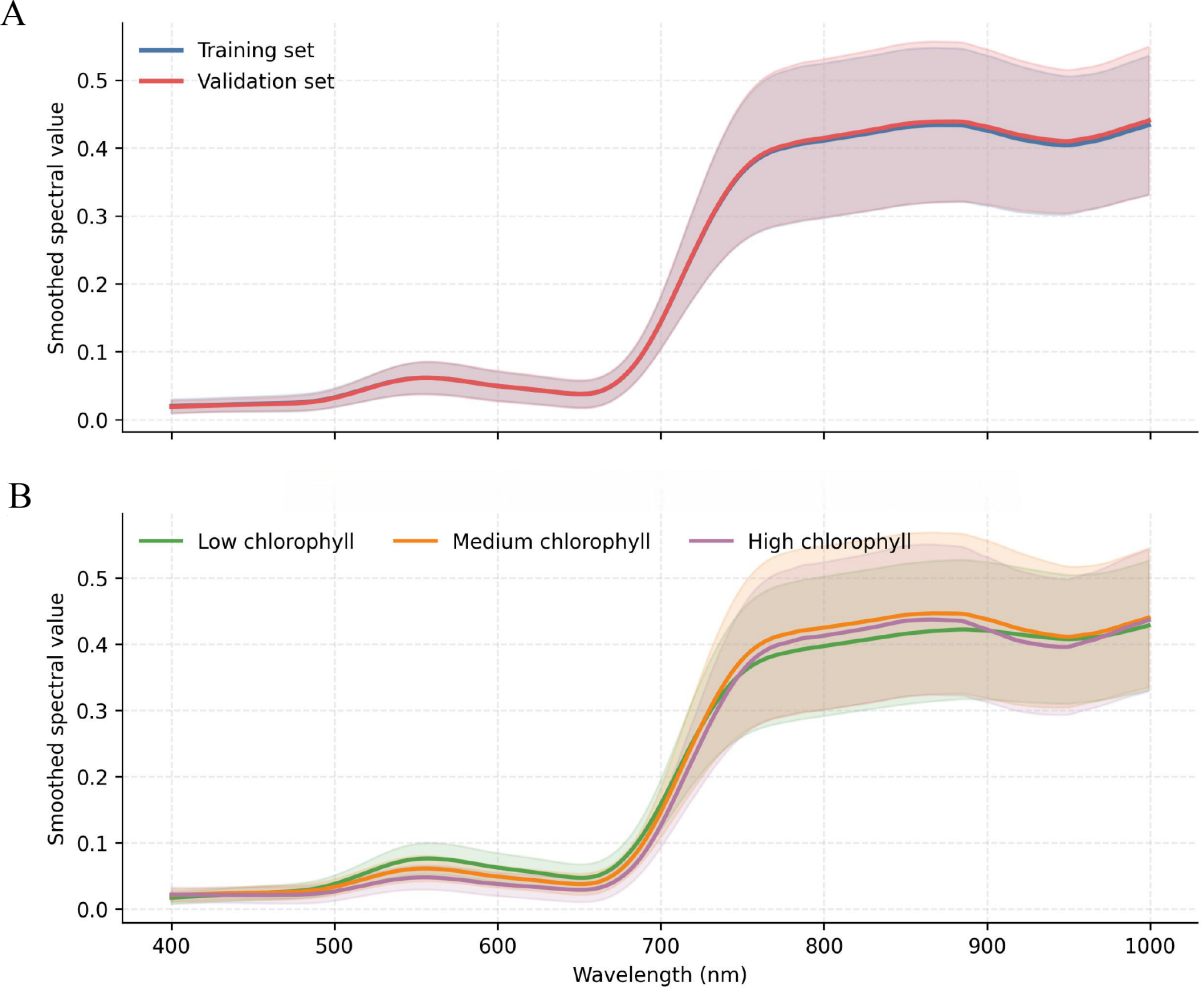
Spectral characterization. **(A)** Averaged smoothed spectra of the training and validation sets. **(B)** Mean smoothed spectra stratified by chlorophyll level.

After band trimming and moving-average smoothing, we first examined whether chlorophyll-related information remained visible in the spectra. **Figure 1** presents the averaged smoothed spectra for the training and validation sets and the mean spectra stratified by chlorophyll level. This comparison was used to verify both spectral consistency across the split and physiological separability across chlorophyll levels.

As shown in **Figure 1**, the training and validation sets retained similar smoothed spectral shapes, indicating that preprocessing did not introduce obvious distortion. Samples with low, medium, and high chlorophyll levels also showed systematic differences in the visible absorption region and the red-edge transition zone. These patterns suggest that chlorophyll information is represented by stable spectral structure rather than by isolated wavelengths alone.

#### 2.1.2 Sample construction

**Figure 2:**
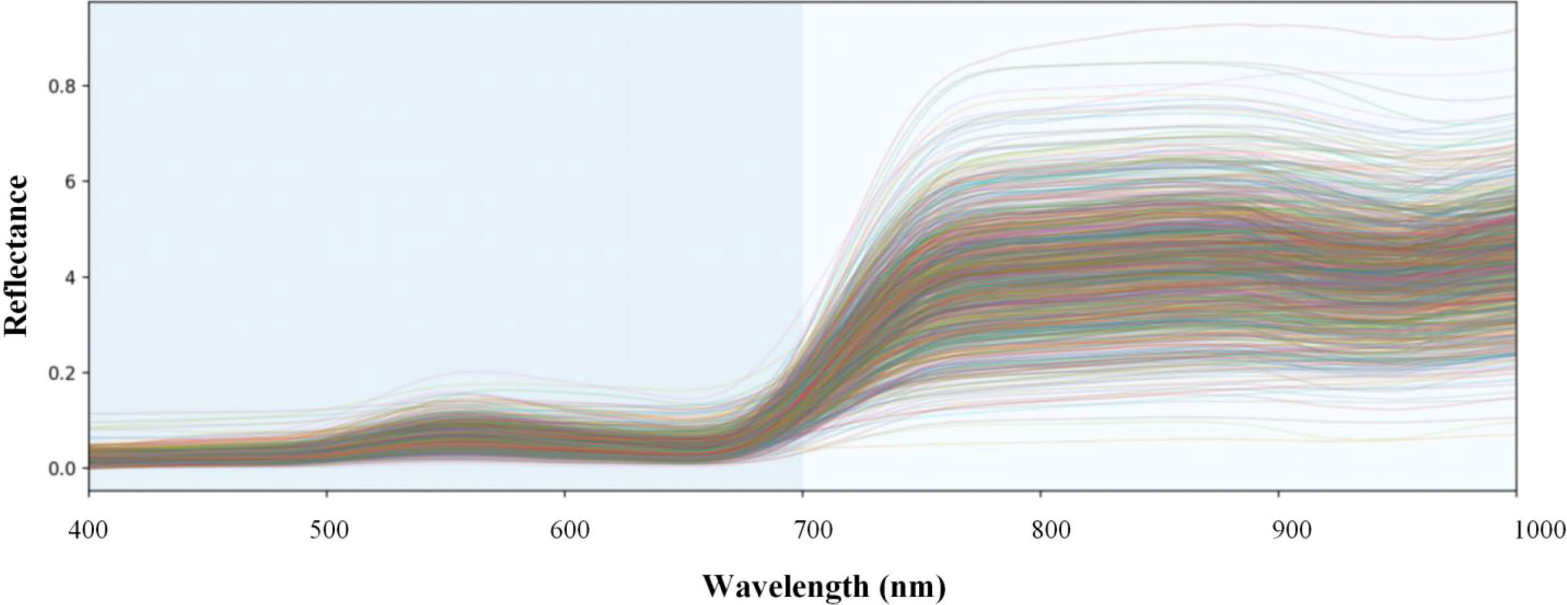
Spectral dataset used for modeling.

After filtering, a total of 1,113 valid samples were retained for modeling. The spectra were cropped to the range of 400–1000 nm, which covers the visible, red-edge, and near-infrared bands most relevant to chlorophyll estimation. Subsequently, a moving average filter with a window size of 63 was applied to smooth the spectra, suppressing local noise while preserving the main spectral shape. This window size was not treated as an independent optimization parameter but rather as an empirical compromise based on the spectral sampling density of this study and the characteristic width of chlorophyll-sensitive features: a smaller window would struggle to effectively suppress high-frequency noise, whereas a larger window might oversmooth the absorption and red-edge structures around 670–750 nm. The processed dataset is shown in **Figure 2**.

**Figure 3:**
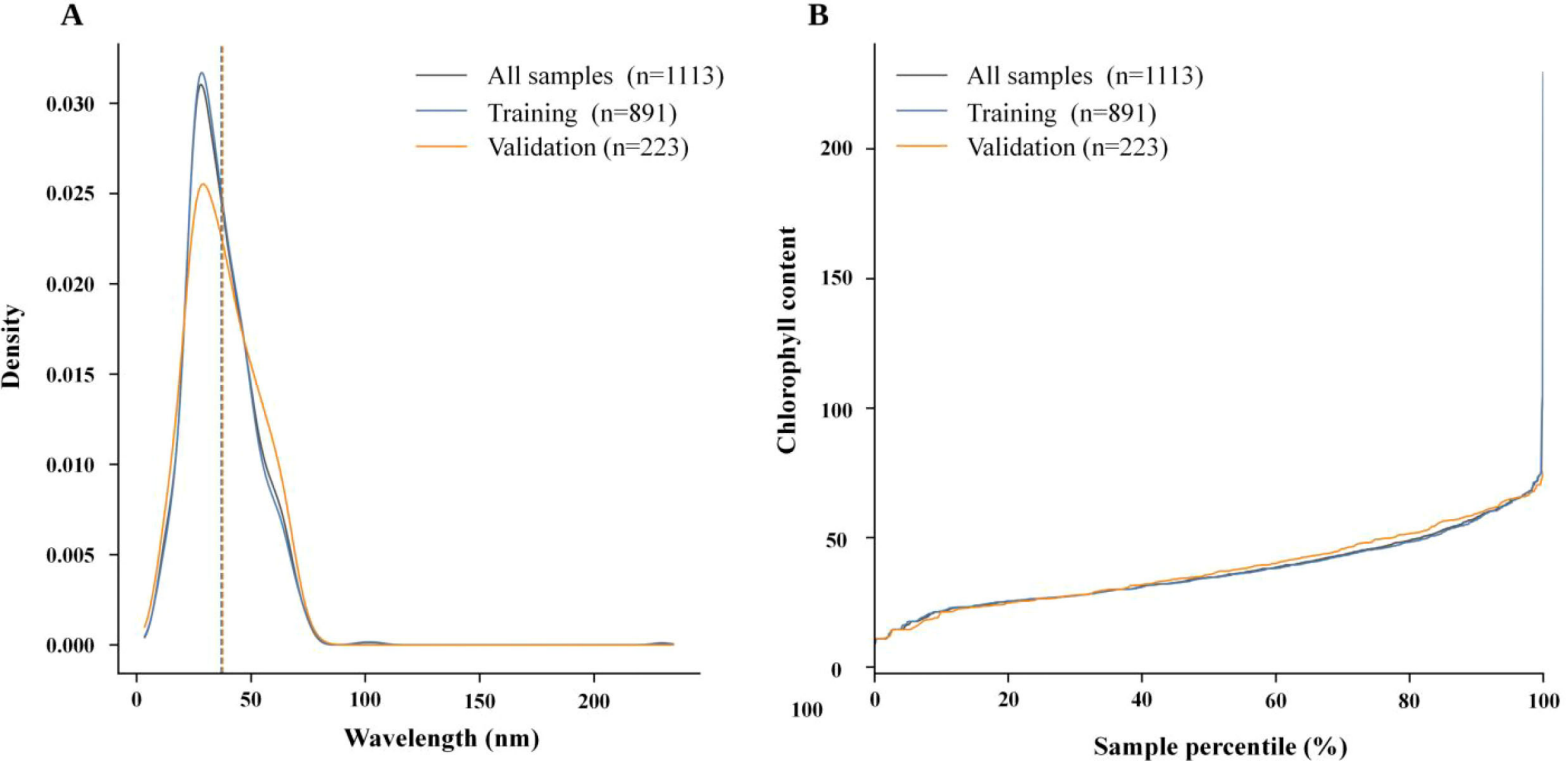
Chlorophyll-value distributions of the full dataset, the filtered modeling dataset, and the train/validation subsets. **(A)** Density distributions of chlorophyll reference values. **(B)** Chlorophyll reference value as a function of sample percentile.

After confirming that the smoothed spectra exhibited interpretable chlorophyll-stratified features, we further examined the sample construction from the perspective of label distribution. **Figure 3** compares the statistical distribution of chlorophyll values among the full dataset, the training set, and the validation set, aiming to assess whether the sample selection and random splitting preserved adequate coverage of the target space. As shown in **Figure 3**, the 1,113 retained samples still covered a wide range of chlorophyll concentrations, and the distributions of the training and validation sets were largely consistent across the main intervals, indicating that the data splitting did not alter the overall structure. Meanwhile, the slightly narrower coverage of the validation set at the high-value end suggests that subsequent interpretation of prediction biases in extreme ranges should be accompanied by more cautious examination using scatter plots and residual plots.

#### 2.1.3 Independent external validation data

Independent external validation data were collected to evaluate the transferability of the model under real-world sampling conditions, with the objective of examining whether the model can maintain valid correlations across different sampling pipelines. This dataset was collected in Aksu on December 21, 2025, comprising a total of 90 tomato leaf samples. Reference chlorophyll values were determined by anhydrous ethanol extraction combined with UV spectrophotometry, and these values were paired with the corresponding spectra to form an external validation set.

#### 2.1.4 Definition and source comparability of the target variable

In this paper, the supervised target is uniformly denoted as the leaf chlorophyll reference value ‘y’. For public datasets, only records that can be uniquely matched with sample identifiers and that serve as continuous chlorophyll reference quantities are retained. For the 90 external samples, reference values are obtained via anhydrous ethanol extraction combined with spectrophotometry. Considering that the public datasets originate from multi-source projects, the comparability between the two types of labels is confined to “continuous reference quantities pointing to the same physiological attribute,” without assuming that full equalization across different sources has been achieved. Consequently, a decline in external accuracy may arise either from spectral distribution shifts or, in part, from differences in reference value measurement chains, sample types, and batch variations. To avoid introducing additional empirical assumptions, this paper interprets the external validation results as a test of transferability under a unified prediction framework.

### 2.2 Methods

#### 2.2.1 Software environment and workflow

**Figure 4:**
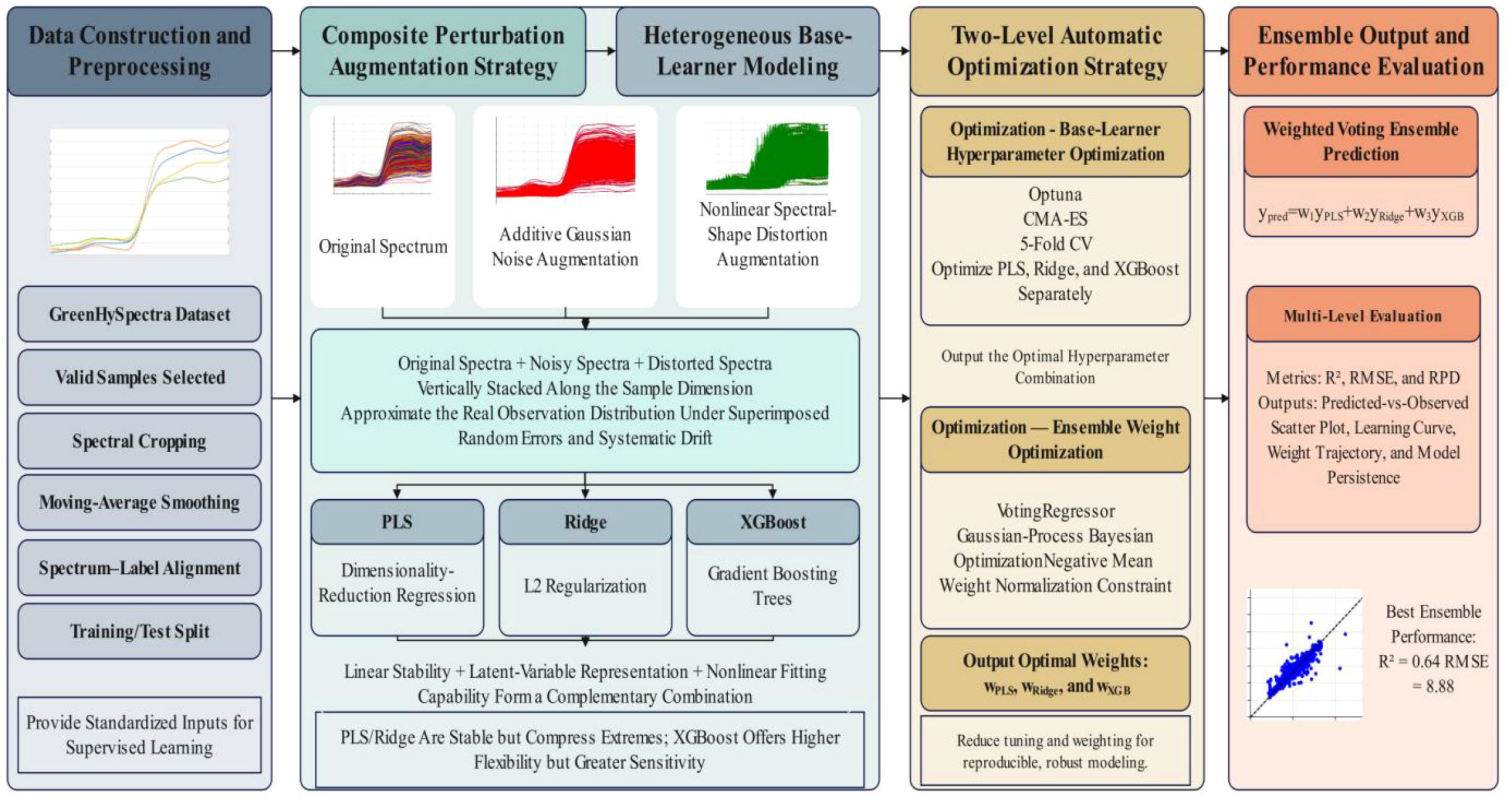
Overall machine-learning regression workflow for hyperspectral chlorophyll estimation.

This study constructs a machine learning regression analysis pipeline based on the Python ecosystem, as illustrated in **Figure 4**. The overall framework is organized as a closed-loop evidence chain encompassing “data processing - feature construction - model training and optimization - performance evaluation - result storage.” This pipeline emphasizes data consistency and reproducibility in high-dimensional spectral modeling and provides a unified experimental baseline for later comparisons of the differential responses to various augmentation strategies and different learners.

All analyses were implemented in Python. The workflow included dataset matching, spectral preprocessing, augmentation of the training set, model training, hyperparameter optimization, weighted ensembling, and performance evaluation. Scikit-learn was used for data partitioning, preprocessing, partial least squares regression, and ridge regression (Pedregosa et al., 2011); XGBoost was used as the nonlinear learner; Optuna with the CMA-ES sampler was used for hyperparameter tuning; and scikit-optimize was used to optimize the ensemble weights. Fixed random seeds were used for all stochastic procedures to improve reproducibility. **Figure 4** summarizes the overall workflow used in this study.

#### 2.2.2 Dataset matching, integration, and type specification

To construct supervised learning samples, this study integrates two types of CSV data: one comprises target files containing sample identifiers and their corresponding chlorophyll values, and the other comprises spectral feature files containing the same identifiers and their reflectance sequences within the 400–1000 nm range. After one-to-one matching, a supervised sample set is established. Moving average smoothing is applied to attenuate random noise and baseline drift while preserving the main spectral profile, thereby enhancing the stability of spectral inputs in subsequent modeling.

#### 2.2.3 Data augmentation and train–validation split

Data augmentation was used as a form of input-space regularization under small-sample conditions. Two perturbations were considered: additive Gaussian noise to emulate random measurement noise, and smooth spectral warping to emulate wavelength-wise shape drift caused by calibration offsets, illumination changes, or sample-state differences (Shorten and Khoshgoftaar, 2019; Nalepa et al., 2019; Rinnan et al., 2009). The perturbation magnitudes were intentionally kept small so that the augmented spectra would remain physiologically plausible. The Gaussian-noise magnitude was searched within 0.001–0.008, whereas the warp magnitude was searched within 0.01–0.07.

The original 1,113 matched samples were first divided into a training set and a validation set using train test split with a 70/30 ratio and random state = 42. Data augmentation was then applied only to the training set. In each experiment, the training data consisted of the original training spectra together with the augmented counterparts generated under the selected setting(s). For single-factor experiments, the original training spectra were combined with either noise-augmented variants or warp-augmented variants, and the corresponding chlorophyll labels were copied to the new samples. For composite augmentation, both perturbations were applied to the same original training spectrum within one augmentation pipeline to generate an additional composite variant, and the original chlorophyll label was retained for that variant. The composite setting was evaluated over 16 noise–warp combinations spanning the pre-specified ranges. Each candidate noise–warp pair was tested by reconstructing the augmented training set under that combination, whereas the validation set remained unchanged. This design prevents augmented variants of the same original sample from appearing in both partitions and therefore avoids information leakage.

For single-factor experiments, the augmented training set consisted of the original spectra and one additional set of perturbed spectra generated by the selected augmentation. For the composite setting, the augmented training set consisted of the original spectra and one additional set of spectra generated by sequentially applying both perturbations. In all cases, the labels of the augmented samples were copied from the corresponding original samples.

**Figure 5:**
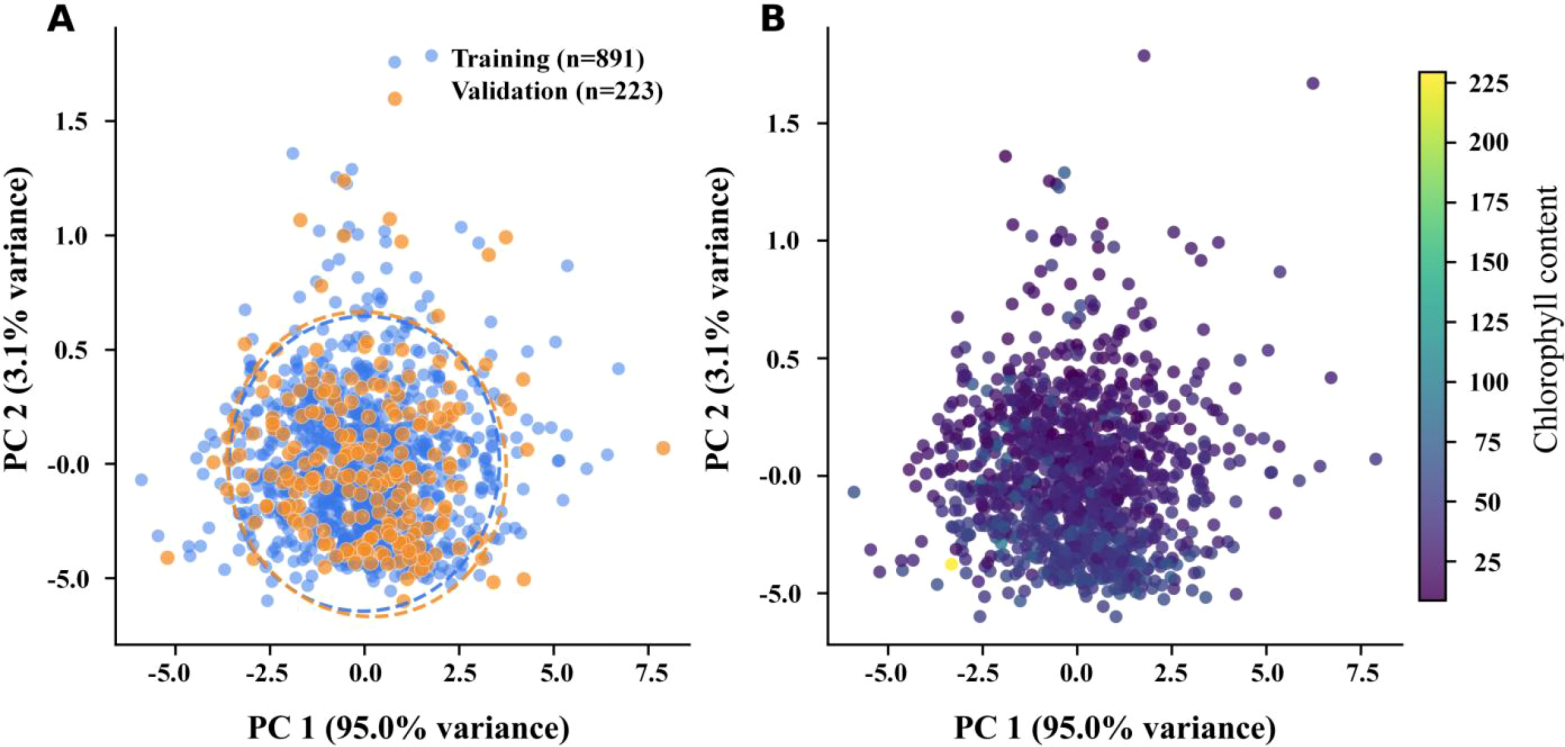
Principal component score plots of the spectral space. **(A)** Training and validation subsets. **(B)** Samples colored by chlorophyll value.

To verify that the split preserved the main spectral structure, the training and validation samples were projected into PCA space, as shown in **Figure 5**.

**Figure 5** indicates that the two subsets overlapped strongly along the primary spectral variation directions. Although some sparsity differences remained near the boundaries, the overall coverage pattern was similar. This suggests that the split preserved the main spectral coverage, but the PCA projection should be interpreted only as a descriptive check rather than as proof that partition-related bias was absent.

### 2.3 Learner configuration

#### 2.3.1 Base learner configuration

This study used three complementary base learners: XGBoost, partial least squares (PLS) regression, and ridge regression. XGBoost was used to capture nonlinear relationships and interaction effects between spectral bands and chlorophyll (Chen and Guestrin, 2016). PLS was used to extract latent variables that maximize covariance between the spectra and the target, making it well suited to high-dimensional collinear data (Wold et al., 2001). Ridge regression was used as a regularized linear model to stabilize coefficients under multicollinearity (Hoerl and Kennard, 1970). Together, these learners provide complementary modeling biases: nonlinear fitting from XGBoost, covariance-based dimensionality reduction from PLS, and stable linear shrinkage from ridge regression.

#### 2.3.2 Hyperparameter configuration and model ensemble

Optuna was employed to search the hyperparameter space of each base learner (Akiba et al., 2019). Objective functions were defined separately for XGBoost, PLS, and ridge regression, and a five-fold cross-validation was performed on the training set, with the maximization of the average coefficient of determination (R²) across folds as the optimization objective. The augmentation parameter ranges have been specified in the Methods section: Gaussian noise was searched within the range of 0.001–0.008, warping magnitude within 0.01–0.07, and for composite augmentation, 16 combinations of noise–warping pairs were evaluated within these ranges. The hyperparameter optimization objective is defined in Equation 1.

(1) $$\underset{\text{θ}}{\text{max}}\frac{1}{5}\sum_{k=1}^{5}R^{2}(y^{\left(k\right)},{\hat{y}}^{\left(k\right)}(\text{θ}))$$

Where (θ) denotes the hyperparameter space to be optimized for the respective models.

**Figure 6:**
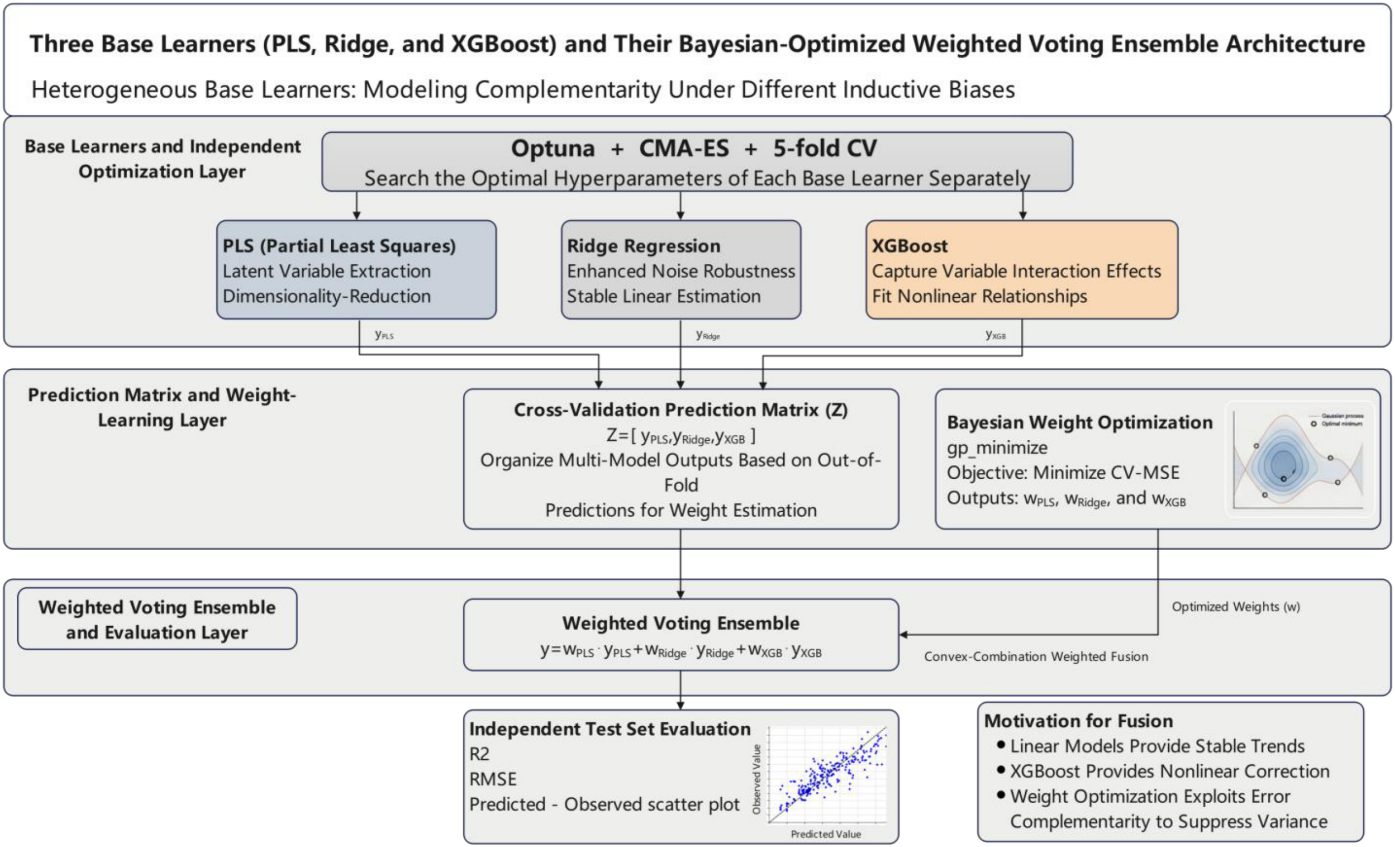
Structure of the three base learners and the Bayesian-optimized weighted voting ensemble.

The CMA-ES sampler (implemented as CmaEsSampler) was adopted because it is effective for continuous hyperparameter optimization (Hansen and Ostermeier, 2001). Each model was optimized for 30 trials, and the best hyperparameter combination was retained for ensemble construction. The structure of the three base learners and the Bayesian-optimized weighted voting ensemble is shown in **Figure 6**.

Upon obtaining the three optimally configured base learners, a weighted ensemble prediction model was constructed using the VotingRegressor. Let the predictions of the three base models on a given sample be denoted as (),(), and (), respectively. The ensemble output is defined as a weighted linear combination. The weighted ensemble prediction is defined in Equation 2.

(2) $$\hat{\text{y}}=\sum_{m=1}^{3}w_{m}{\hat{w}}_{m},w_{m}\geq 0,\sum_{m=1}^{3}w_{m}=1$$

After base-learner tuning, their predictions were combined with a weighted voting ensemble. For a given sample, the ensemble prediction was defined as ŷ_ens_ = w_1_ŷ_XGB_ + w_2_ŷ_PLS_ + w_3_ŷ_Ridge_, subject to w_i_ ≥ 0 and Σ_i_ w_i_ = 1. The weight vector was optimized with gp minimize from scikit-optimize (Head et al., 2021). The objective was the 3-fold cross-validated mean squared error on the training set, so the procedure minimized internal prediction error while respecting the convex-combination constraint (Jones et al., 1998; Snoek et al., 2012).

### 2.4 Model evaluation

Upon completion of model training, performance was evaluated from three aspects: goodness of fit, prediction error, and stability. The coefficient of determination (R²) and root mean square error (RMSE) were computed on both the training set and the validation set. Furthermore, the average performance of the model on unseen data was assessed through cross-validation using R² and RMSE. R² represents the model’s ability to explain variance, while RMSE quantifies the average prediction error in the original chlorophyll units. Together, these metrics are used to compare the models in terms of fitting quality, predictive accuracy, and generalization capability. The coefficient of determination and root mean square error are defined in Equation 3.

(3) $$R^{2}=1-\frac{\sum_{i=1}^{n}{\left(y_{i}-\hat{y}\right)}^{2}}{\sum_{i=1}^{n}(y_{i}-{\bar{y}}_{i}},RMSE=\sqrt{\frac{1}{n}\sum_{i=1}^{n}(y_{i}-\hat{y}}{)}^{2}$$

## 3 Results

Model performance was evaluated using the coefficient of determination (R²) and the root mean square error (RMSE) on the training set, the validation set, and the external validation set. R² measures the proportion of variance explained by the model, whereas RMSE measures the average magnitude of prediction error in the original chlorophyll units. Cross-validation results on the training set were also recorded to support internal model comparison.

**Figure 7:**
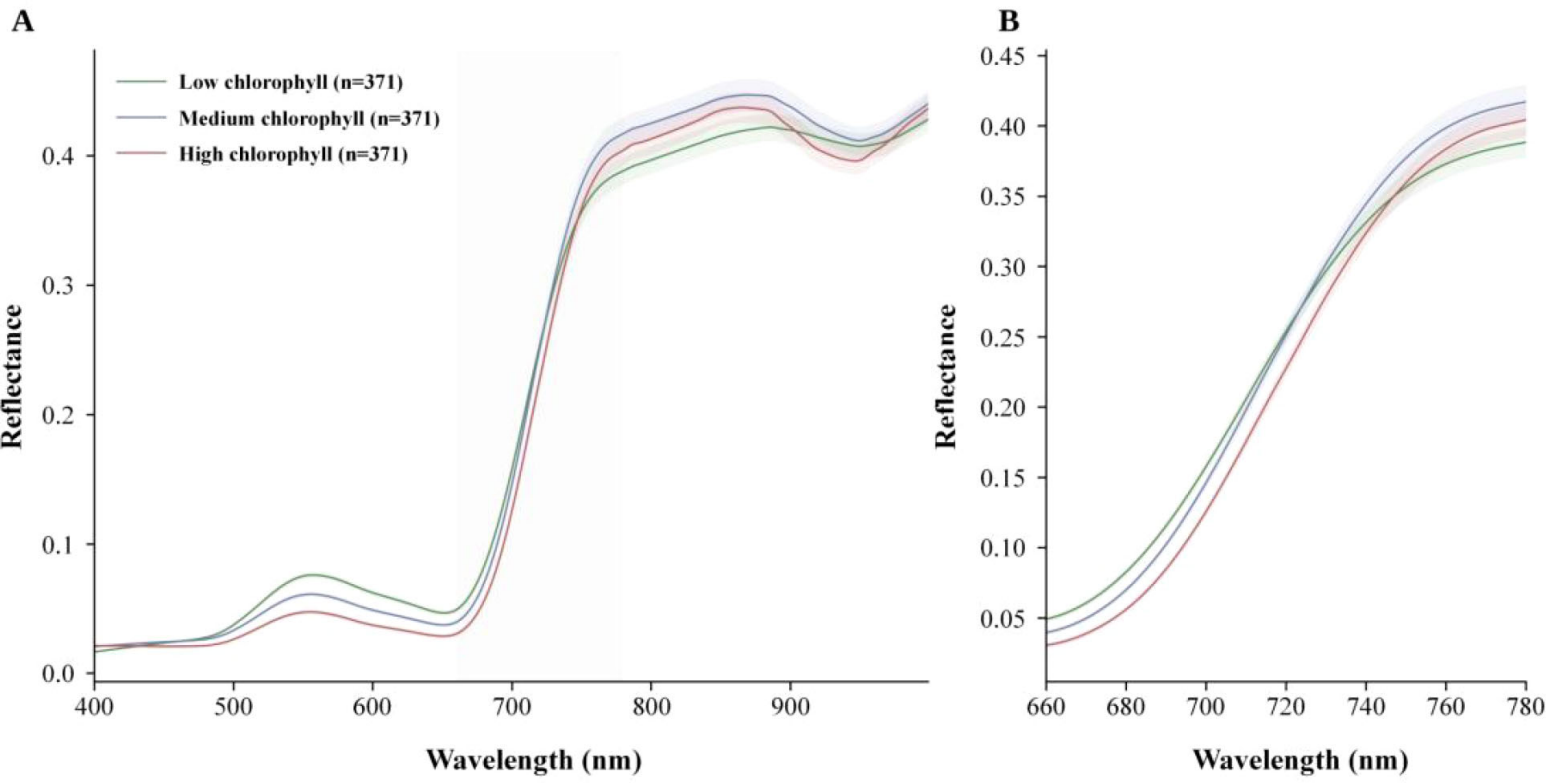
Mean spectra stratified by chlorophyll level. **(A)** Full spectral range from 400 to 1000 nm. **(B)** Enlarged red-edge region from 660 to 780 nm.

**Figure 8:**
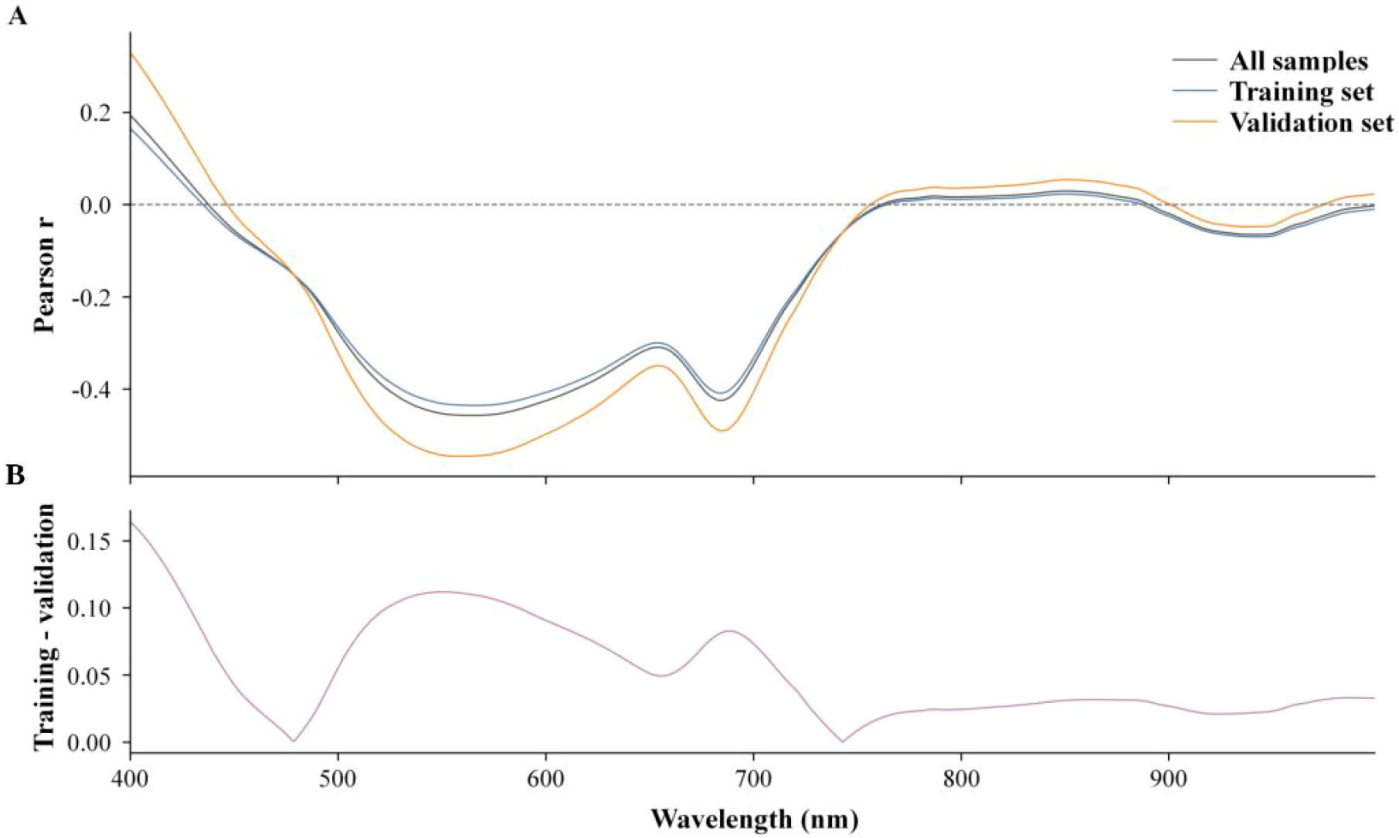
Wavelength-wise correlation profile between reflectance and chlorophyll. **(A)** Pearson correlation profiles for all samples, the training set, and the validation set. **(B)** Difference between the training and validation correlation profiles.

**Figure 9:**
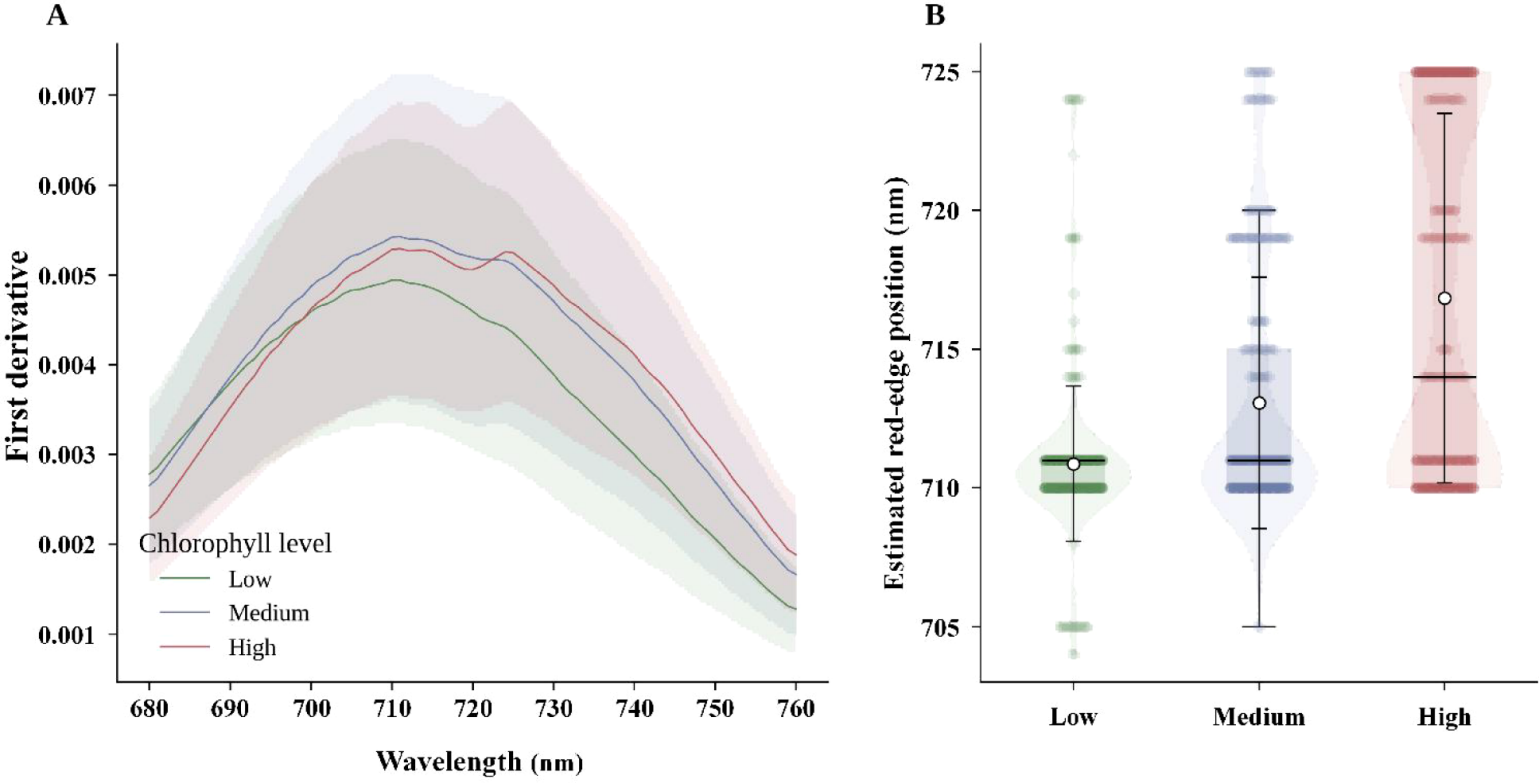
Red-edge response across chlorophyll levels. **(A)** First-derivative spectra for low, medium, and high chlorophyll levels. **(B)** Estimated red-edge position distributions for the three chlorophyll levels.

**Figure 10:**
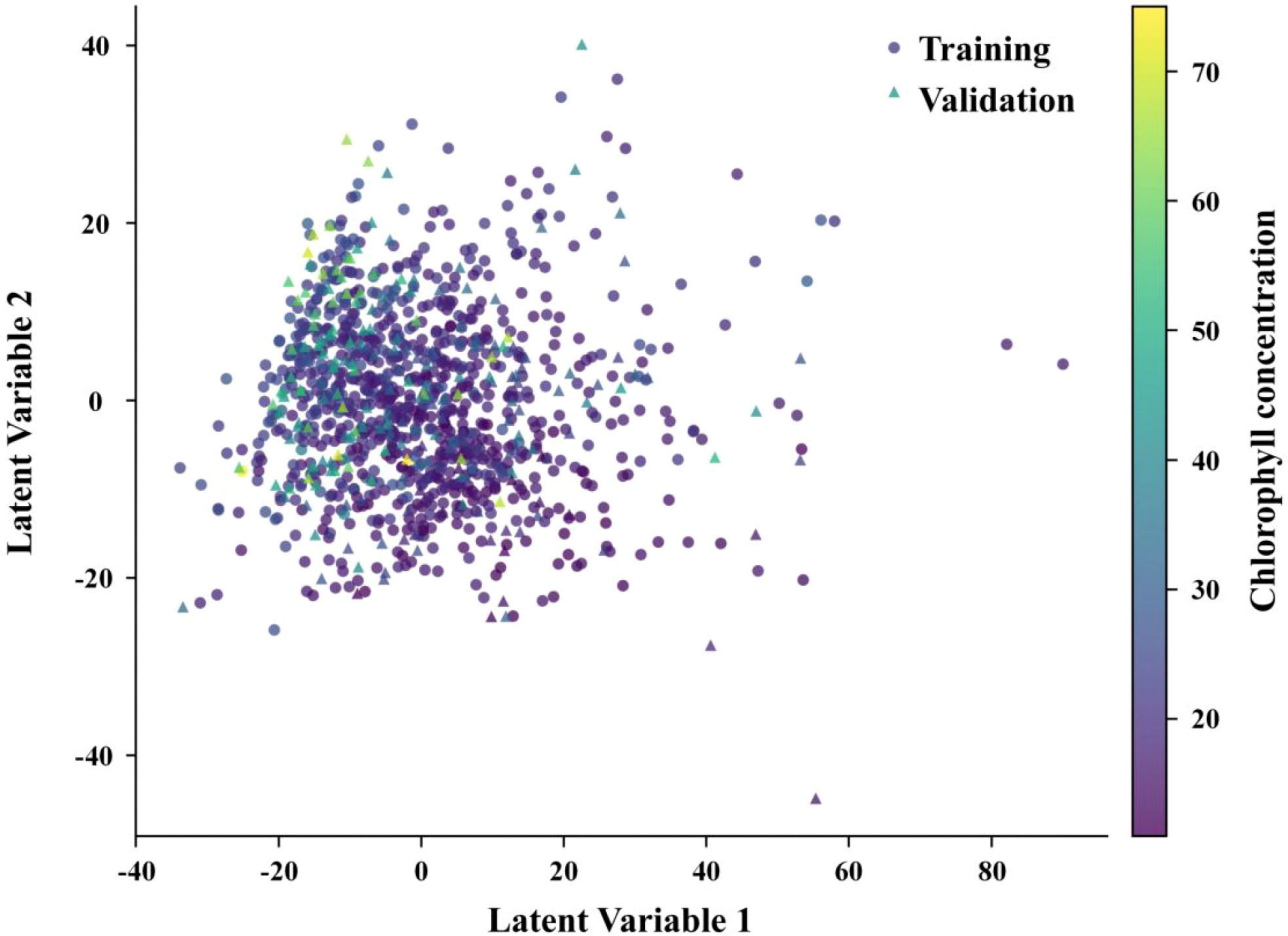
PLS latent-space distribution under the raw baseline setting.

To examine how the supervised latent variable structure organizes the sample distribution, **Figure 10** presents the score space of the first two PLS latent variables. The figure shows that samples are arranged along a chlorophyll-related gradient in the latent variable space, indicating that the primary covariance structure extracted by PLS is physiologically meaningful.

### 3.1 Performance analysis of data augmentation

After confirming that the data inherently exhibit a stable spectral–chlorophyll structure, the next step is to focus on reducing estimation variance and enhancing model robustness to acquisition perturbations under small-sample conditions. To this end, within a rigorous training–validation separation framework, noise injection and nonlinear spectral shape perturbations were applied to the training samples, with the unaugmented model serving as a baseline control. The corresponding results are presented in **Table 1**.

**Table 1:** Baseline performance of individual models without augmentation evaluated on the validation set (single fixed-seed split).

Model	R²	RMSE
PLS	0.4724	11.0949
Ridge	0.4514	11.3137
XGB	0.4163	11.6695

**Table 1** shows that under the condition without augmentation, the RMSE values of the three base learners were all approximately 11, indicating that relying solely on the original training distribution still leaves considerable room for improvement in generalization error. Therefore, the subsequent augmentation experiments focus not merely on whether a particular parameter point achieves a local optimum, but rather on whether the augmentation strategy can consistently reduce error levels across a wider range and improve model robustness.

#### 3.1.1 Noise augmentation

As shown in **Table 2**, noise augmentation provided consistent improvements across all three models within a relatively wide intensity range. Using RMSE as the primary metric, the average reductions for PLS, ridge regression, and XGBoost were 11.62%, 14.55%, and 17.14%, respectively, while the maximum reductions reached 13.14% for PLS at noise = 0.002, 15.55% for ridge regression at noise = 0.001, and 20.92% for XGBoost at noise = 0.008. However, when the noise intensity increased to 0.007, the RMSE of XGBoost deteriorated markedly to 10.6996, suggesting that excessive perturbation weakened the physical plausibility of the augmented spectra. These results indicate that noise augmentation should be applied within a low-to-moderate intensity range rather than selected solely on the basis of a single optimum. Overall, noise augmentation improved all three models across most tested settings and showed relatively small performance fluctuations, indicating a relatively broad stable region for practical use.

**Table 2:** Performance of individual models under Gaussian noise augmentation evaluated on the validation set (single fixed-seed split).

Noise augmentation	PLS		Ridge		XGB	
	R^2^	RMSE	R^2^	RMSE	R^2^	RMSE
0.001	0.5614	9.8003	0.5831	9.5542	0.5672	9.735
0.002	0.5758	9.6372	0.5764	9.631	0.5616	9.7981
0.003	0.5608	9.8068	0.5787	9.6051	0.5972	9.3911
0.004	0.56	9.816	0.5766	9.6291	0.5688	9.7169
0.005	0.5618	9.7956	0.5753	9.6438	0.5141	9.2547
0.006	0.5566	9.8539	0.5703	9.6997	0.5853	9.5294
0.007	0.555	9.8718	0.5625	9.7875	0.4772	10.6996
0.008	0.5553	9.8676	0.5624	9.7885	0.6011	9.2279

#### 3.1.2 Smooth spectral warping

In contrast to noise augmentation, which mainly perturbs reflectance values, smooth spectral warping acts more directly on spectral shape. Therefore, it holds the potential to compensate for distribution shifts while also being more prone to disrupting physical consistency under strong perturbations. **Table 3** reveals a consistent trend across the three individual models: moderate augmentation yields benefits, whereas excessive augmentation leads to degradation. Notably, XGBoost exhibited its best performance at warp = 0.04 in the present experiment, whereas the linear models (PLS and ridge regression) showed a greater tendency for error rebound under higher distortion intensities.

**Table 3:** Performance of the individual models under smooth spectral warping augmentation (warp) on the validation set (R² and RMSE).

Warp augmentation	PLS		Ridge		XGB	
	R^2^	RMSE	R^2^	RMSE	R^2^	RMSE
0.01	0.5604	9.8109	0.5615	9.7989	0.5266	10.181
0.02	0.5538	9.8845	0.5579	9.8391	0.5656	9.7534
0.03	0.5473	9.956	0.5528	9.8961	0.5475	9.9539
0.04	0.5431	12.068	0.5433	9.9896	0.5727	9.6733
0.05	0.4918	10.549	0.5387	10.0507	0.5470	9.9601
0.06	0.4876	10.592	0.5268	10.1795	0.5165	10.2890
0.07	0.4845	10.6244	0.5306	10.1382	0.5306	10.1382

**Figure 11:**
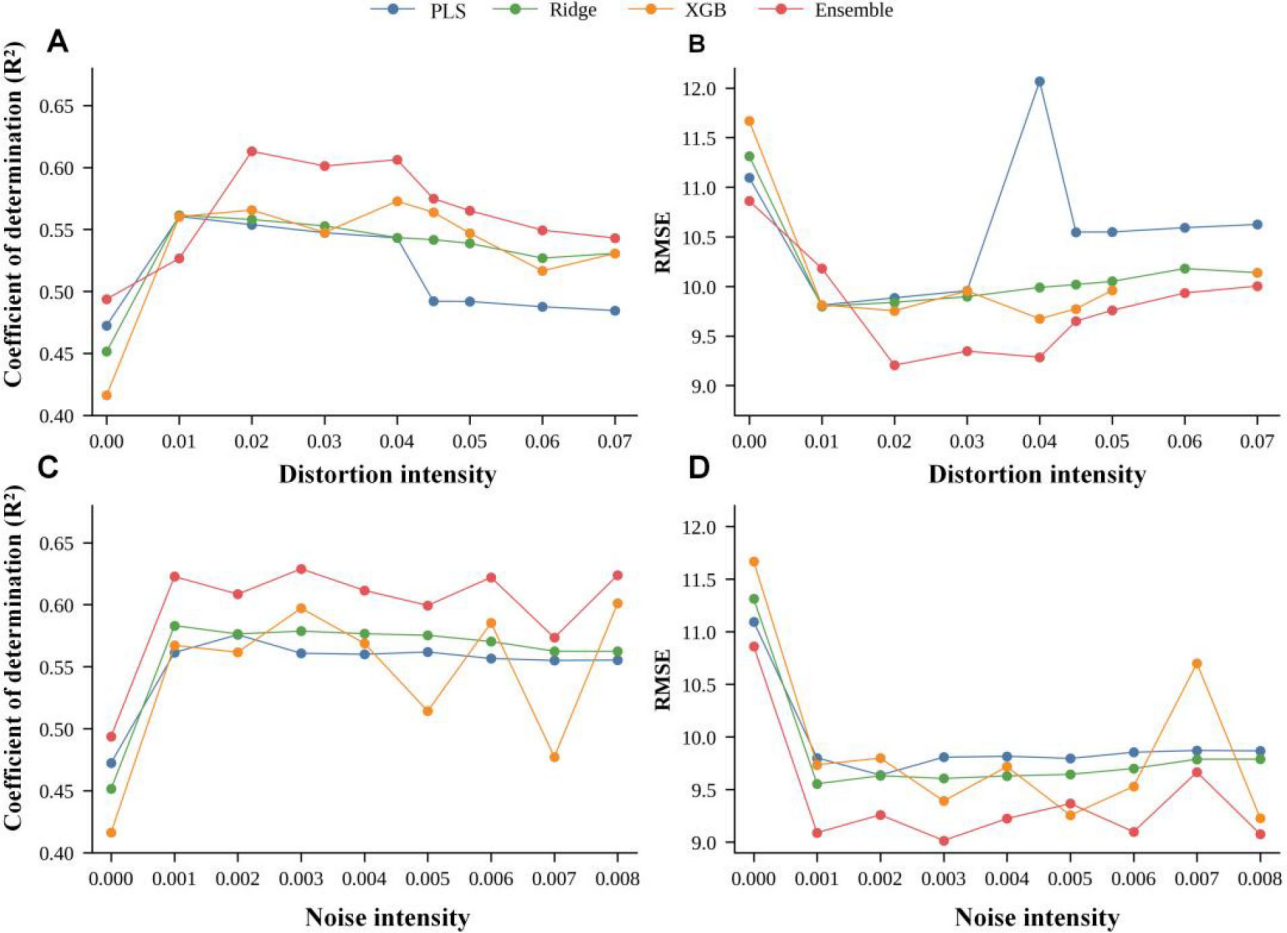
Single-factor augmentation response curves. **(A)** PLS. **(B)** Ridge. **(C)** XGBoost. **(D)** Weighted ensemble.

To convert the discrete numerical values in the table into continuous trends, **Figure 11** plots the performance trajectories under noise augmentation and smooth spectral warping within the same coordinate system. From **Figure 11**, it can be seen more intuitively that noise augmentation yields a broader performance plateau, whereas the beneficial region of smooth spectral warping is narrower and more sensitive to perturbation intensity. In other words, noise augmentation behaves more like a robust variance suppression term, while smooth spectral warping acts as a distribution shift compensation term, whose benefit depends more strongly on the degree of consistency with the true spectral drift.

From **Figure 11**, it can be more intuitively observed that noise augmentation forms a relatively broad performance plateau, whereas smooth spectral warping, despite achieving a higher peak, is more sensitive to intensity variations. In other words, noise augmentation functions more as a robust variance suppressor, while smooth spectral warping behaves more like a distribution shift compensator—its benefits are more dependent on the degree of alignment with actual spectral shape deviations.

#### 3.1.3 Composite augmentation

**Table 4** summarizes the predictive performance of the three base learners (PLS, ridge regression, and XGBoost) under composite augmentation (noise × warp). All experiments were conducted within a strict training–validation separation framework to avoid information leakage introduced by data augmentation. Across the 16 composite-augmentation combinations (noise = 0.001–0.004, warp = 0.01–0.025), the average RMSE reductions relative to the non-augmented baseline were 10.61% for PLS, 12.85% for ridge regression, and 17.54% for XGBoost. The maximum reductions reached 12.53% for PLS at noise = 0.001 and warp = 0.01, 14.15% for ridge regression at noise = 0.001 and warp = 0.01, and 21.69% for XGBoost at noise = 0.002 and warp = 0.025. These results indicate that composite augmentation consistently reduced prediction error across the tested combinations, with the strongest improvement observed for XGBoost among the three individual models.

**Table 4:** Performance of individual models under composite augmentation (noise × warp) evaluated on the validation set (single fixed-seed split).

Composite augmentation		PLS		Ridge		XGB	
Noise	Warp	R^2^	RMSE	R^2^	RMSE	R^2^	RMSE
0.001	0.01	0.5698	9.7052	0.5692	9.7127	0.5831	9.5547
0.001	0.015	0.5493	9.9339	0.5563	9.8566	0.5944	9.4237
0.001	0.02	0.5488	9.9401	0.5553	9.8679	0.5903	9.4714
0.001	0.025	0.5463	9.967	0.5526	9.8978	0.5694	9.7106
0.002	0.01	0.5521	9.9034	0.5590	9.8267	0.6178	9.1477
0.002	0.015	0.5518	9.907	0.556	9.8605	0.5207	10.2442
0.002	0.02	0.5507	9.919	0.5556	9.8646	0.6147	9.1847
0.002	0.025	0.5463	9.9676	0.5523	9.909	0.6186	9.1387
0.003	0.01	0.5513	9.9124	0.558	9.8383	0.5580	9.8383
0.003	0.015	0.5534	9.8893	0.557	9.849	0.5790	9.6015
0.003	0.02	0.5487	9.941	0.554	9.8828	0.5814	9.5734
0.003	0.025	0.5481	9.9473	0.5515	9.9104	0.5674	9.7324
0.004	0.01	0.5509	9.9171	0.5569	9.8505	0.5029	10.4331
0.004	0.015	0.5514	9.9115	0.5553	9.8675	0.5689	9.7159
0.004	0.02	0.5481	9.9474	0.5538	9.8842	0.5624	9.7890
0.004	0.025	0.5457	9.9736	0.5535	9.8879	0.5963	9.4015

**Figure 12:**
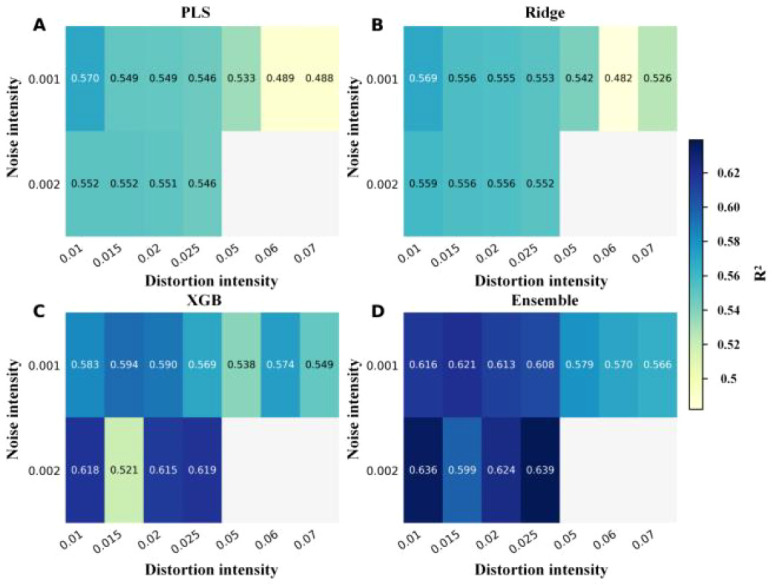
Validation R² heatmaps under composite augmentation. **(A)** PLS. **(B)** Ridge. **(C)** XGBoost. **(D)** Weighted ensemble.

**Figure 13:**
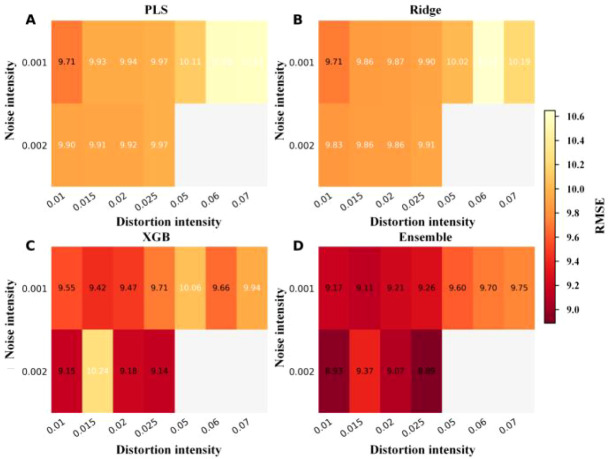
Validation RMSE heatmaps under composite augmentation. **(A)** PLS. **(B)** Ridge. **(C)** XGBoost. **(D)** Weighted ensemble.

Given that single-factor augmentation has already demonstrated clear beneficial ranges, we further investigate whether stable synergistic effects exist when noise and distortion are applied in combination. To this end, **Figures 12** and **13** present the overall performance landscape of composite augmentation using two-dimensional contour maps of R² and RMSE, rather than merely reporting individual optimal combinations.

Examining the two figures together reveals that the high-performance regions are not isolated peaks but rather form continuous plateaus around noise≈0.001–0.003 and warp≈0.01–0.025: the high R² areas in **Figure 12** correspond to the low RMSE zones in **Figure 13**, indicating that moderate composite perturbations can simultaneously improve explanatory power and reduce error levels. This plateau-like characteristic suggests that parameter selection can prioritize robust regions rather than relying on isolated point optima.

### 3.2 Model performance

#### 3.2.1 Base learners

**Figure 14:**
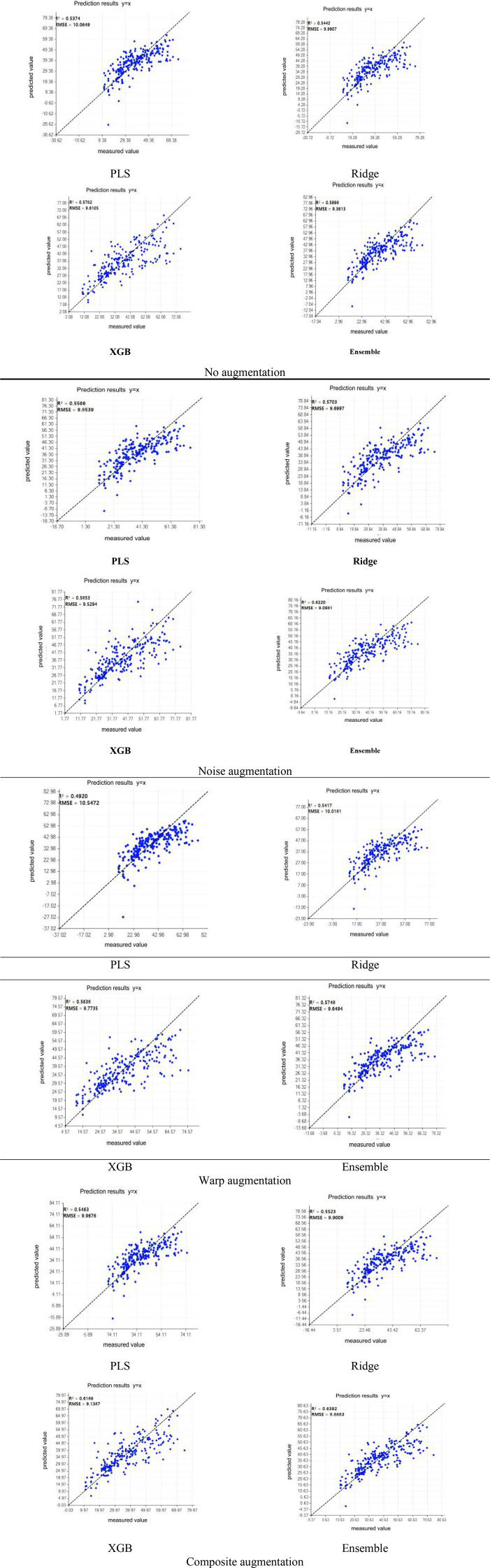
Predicted-versus-measured scatter plots for the base learners and the weighted ensemble under different augmentation scenarios. Panels are arranged by model and augmentation setting as labeled in the image.

**Figure 14** summarizes the relationship between predicted and measured chlorophyll values under four augmentation scenarios. Overall, XGBoost and the ensemble model lie closer to the 1:1 line than the linear models, particularly in the mid-to-high value region. Under the optimal composite augmentation setting, the ensemble model achieves R² = 0.6392 and RMSE = 8.8883 on the validation set; the best single model is XGBoost under noise = 0.002 and warp = 0.025 (R² = 0.6186, RMSE = 9.1387). On this basis, the ensemble model further reduces RMSE by 2.74% and increases R² by 0.0206 compared to the best single model.

Examining the scatter distributions, the point cloud for PLS is relatively concentrated in the mid-value range. However, it exhibits systematic underestimation in the high-value range (with more points falling below the y=x line) and a tendency towards relative overestimation in the low-value range. Although PLS reduces dimensionality through latent components, it remains fundamentally a linear model: it projects the original spectral space onto a low-dimensional latent space before performing linear regression. In scenarios characterized by high dimensionality, strong multicollinearity (typical of hyperspectral data), and limited effective sample sizes, regularization and dimensionality reduction significantly reduce the model’s “effective degrees of freedom.” This leads to a classic phenomenon: to minimize the overall mean squared error (particularly the error in the densely populated mid-range), the model tends to shrink predictions towards the overall mean—a behavior known as “regression to the mean.” Further analysis of the error patterns in each subplot reveals that PLS and Ridge are relatively stable in the mid-range but still show significant compression for high-value samples. XGBoost, by contrast, is better able to recover nonlinear variations, thus reducing bias at the high end. The ensemble model, while retaining XGBoost’s advantage in nonlinear correction, leverages the stable trends of the linear models to mitigate scattered outliers and systematic drift. This graphical evidence of progressive improvement is consistent with the performance improvements reported in the corresponding tables.

#### 3.2.2 Ensemble model performance

**Figure 15:**
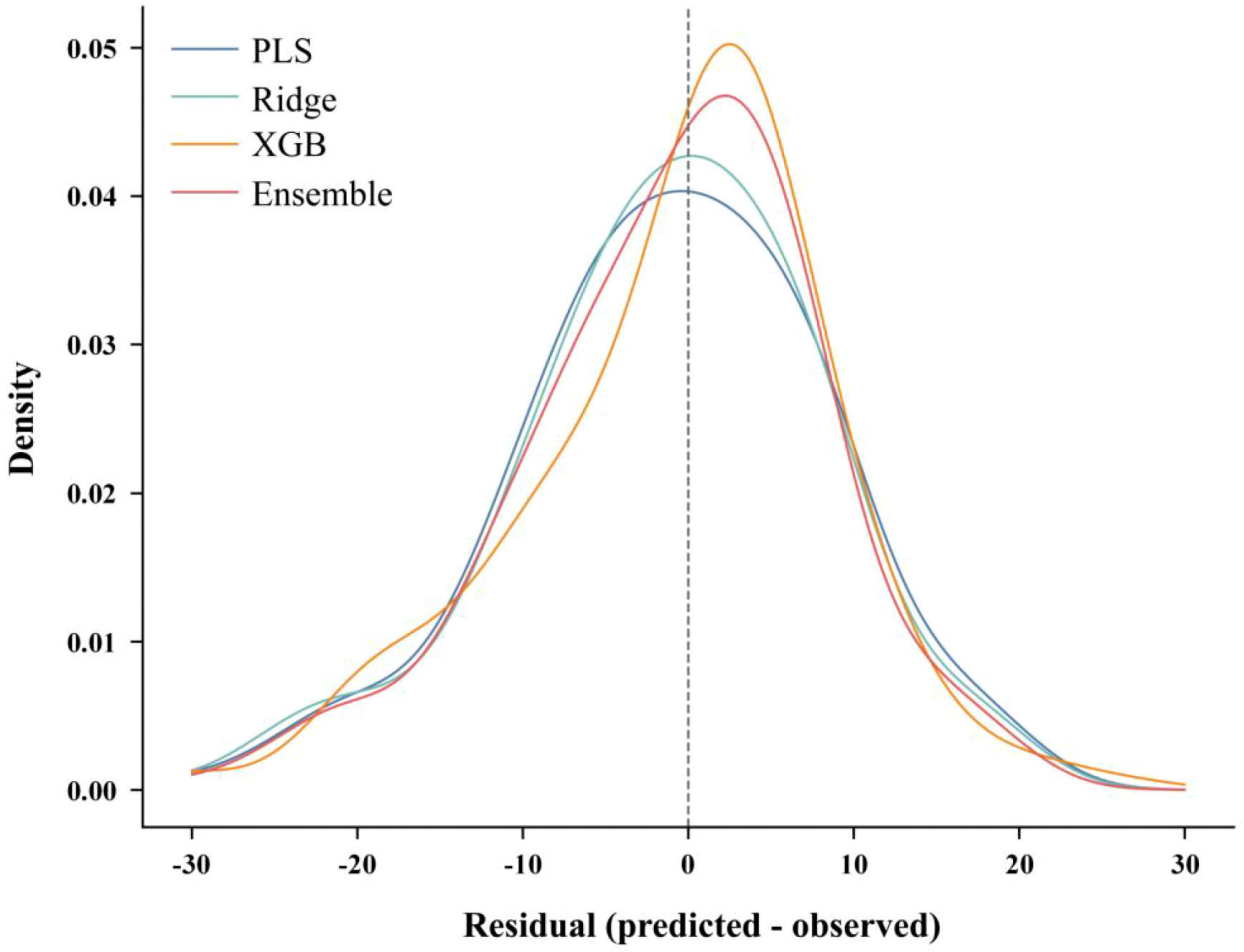
Residual density curves for the best overall model setting.

The parity plots described above illustrate the overall fitting relationships, with the ensemble model exhibiting the closest alignment to the y=x line and the smallest overall dispersion (lowest RMSE). This generally arises from two optimization mechanisms: the partial cancellation of errors among different models, and the combination of stable trend provision by linear models with nonlinear correction by XGBoost, which together reduce systematic underestimation and overestimation after fusion. XGBoost demonstrates its advantage as a nonlinear model by capturing nonlinearities and interaction terms in the spectrum–chlorophyll relationship. Among the two linear models, ridge regression showed slightly better stability than PLS, although the difference between them was limited. However, the ensemble model consistently achieves stable improvements over the best individual model, indicating that the errors of the three models are not entirely aligned in direction and that a complementary space exists for exploitation. Therefore, after confirming the overall optimality of the ensemble model, it is necessary to further examine whether its errors are more concentrated and whether interval-specific biases persist. Based on this consideration, **Figure 15** compares the residual density distributions of each model under the optimal augmentation scenario.

**Figure 15** clarifies whether residuals are tightly concentrated around zero or dispersed with heavy tails; in practical terms, a narrower and more centered profile indicates better-calibrated predictions.

Across all 16 composite augmentation combinations, the ensemble model outperformed the optimal single model within each group, with the predicted-versus-measured scatter relationships of PLS, ridge regression, XGBoost, and the voting ensemble model on the validation set exhibiting a progressive improvement trend. As linear models, PLS and ridge regression demonstrated stable performance but exhibited some degree of extreme-value compression. XGBoost, by contrast, more effectively captured the nonlinear relationships between spectra and chlorophyll, thereby achieving higher explanatory power and lower error. Under the best composite-augmentation setting, the voting ensemble further enhanced fitting consistency (R² = 0.6392, RMSE = 8.8883), reducing RMSE by 2.74% relative to the best individual model, XGBoost, obtained at noise = 0.002 and warp = 0.025 (R² = 0.6186, RMSE = 9.1387). Combined with the results reported in **Tables 2**-**4**, both noise augmentation and smooth spectral warping reduced the error of individual models, with noise augmentation providing more stable improvements and smooth spectral warping achieving higher peak gains on nonlinear models. Composite augmentation yielded consistent improvements across most combinations and significantly enhanced the robustness advantage of the ensemble model relative to single models (average RMSE reduction of 3.71%, average R² improvement of 0.0308), thereby supporting the effectiveness of the “composite augmentation + ensemble learning” synergy in improving generalization performance.

**Figure 16:**
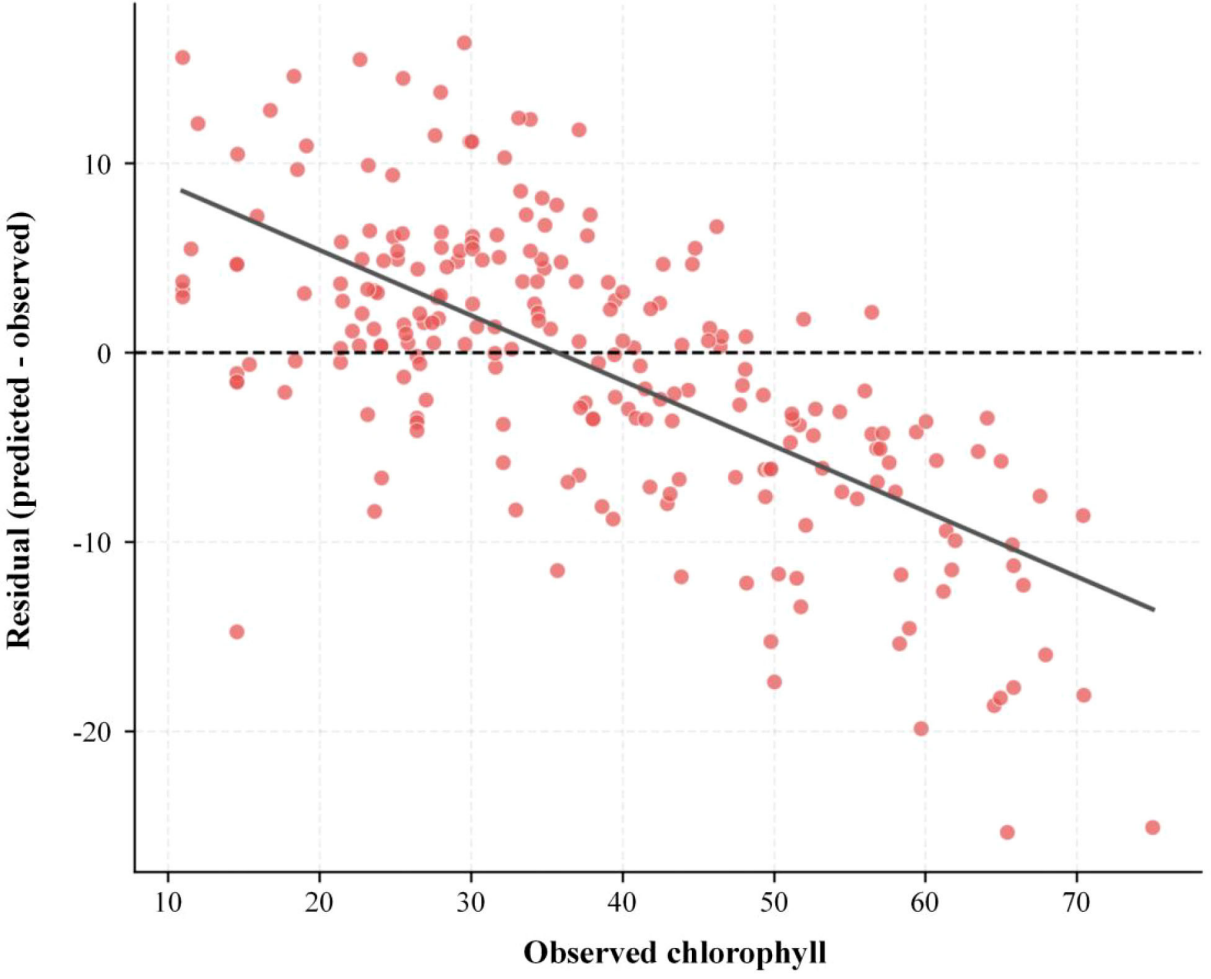
Residuals versus observed chlorophyll values for the best overall model setting.

This improvement likely arose because the three learners produced partly different error patterns across the chlorophyll range: PLS and Ridge provide smooth global trends, while XGBoost compensates for local nonlinearities, enabling the ensemble’s errors to be partially offset across different chlorophyll intervals. To further assess whether this error compression remains stable across the entire target range, we mapped the ensemble model’s residuals against the measured chlorophyll values in **Figure 16**.

**Figure 16** reveals that the residuals are not entirely randomly distributed; a certain downward bias persists in the high-value range, indicating that even the optimal ensemble model, while significantly improving overall error, may still retain some degree of magnitude compression in extreme segments. These residual patterns help explain where the ensemble still underperformed and where its gains were most evident.

#### 3.2.3 Independent external validation results

To further evaluate external applicability under real-world sampling conditions, the optimal ensemble model trained on the public dataset was directly applied to 90 independently collected tomato leaf samples. The model achieved R² = 0.498 and RMSE = 9.801 on the external validation set. Although the accuracy decreased compared to the validation set results, the predicted values remained positively correlated with the measured values, indicating that part of the spectral signal learned by the model can be transferred to independent samples collected under different conditions. It should be emphasized that this performance decline may arise either from spectral distribution shifts or, in part, from differences in sample types, acquisition pipelines, and reference value measurement procedures; therefore, the external results should be understood as a test of cross-source transferability rather than as a strict absolute calibration.

## 4 Discussion

### 4.1 Comparison with previous studies

Existing research on hyperspectral chlorophyll estimation has broadly followed three methodological routes. The first route emphasizes improving spectral inputs through preprocessing, band selection, or feature extraction. The second route reduces variance and exploits complementary predictive behaviors by combining multiple learners. The third route employs more complex deep models to directly learn spatial–spectral features. Due to differences across studies in crop type, observation scale, spectral configuration, sample size, and chlorophyll measurement units, absolute R² and RMSE values from the literature are not suitable for direct horizontal comparison. Therefore, the comparison in this paper focuses more on the logic of improvement.

In the first direction, model performance is improved mainly by refining the spectral input. Sonobe et al. (2020) compared several machine-learning models for tea-leaf chlorophyll estimation, Chen et al. (2020) combined GA with PLSR for rapeseed leaves, and Zhang et al. (2023) used feature-band selection with CatBoost for apple leaves. These studies show that improved spectral representation can enhance chlorophyll estimation, but the overall framework still relies mainly on a single learner.

The second direction emphasizes model integration. Huang et al. (2023) combined PROSAIL-generated samples with stacking, Chen et al. (2024) used integrated feature selection with GBRT, and Guo et al. (2025) evaluated stacking strategies for tea leaves. These studies indicate that heterogeneous integration can reduce prediction error when different learners capture complementary aspects of the spectrum–target relationship.

The third direction uses deep learning to extract more complex features directly from spectral or spatial–spectral data. Zhao et al. (2024) reported strong performance with a 3DCNN–LSTM framework, and Tsuchiya et al. (2025) improved tea-leaf chlorophyll estimation through self-supervised learning. These methods show the potential of deeper feature extraction, but they usually require more data, more complex inputs, or higher computational cost.

Compared with these studies, the present work combines two simpler but complementary strategies. At the input level, low-amplitude Gaussian noise and smooth spectral warping were used to expand the training distribution in a physically plausible way. At the model level, PLS, ridge regression, and XGBoost were combined through weighted ensembling to integrate stable linear trends with nonlinear fitting capacity. The main contribution of this study is therefore not the use of a more complex model, but the joint use of physically constrained augmentation and heterogeneous ensembling for small-sample chlorophyll estimation.

### 4.2 Performance gains from augmentation and ensemble integration

Ablation results indicate that performance improvements stem from both data augmentation and ensemble modeling. Relative to the non-augmented baseline, noise augmentation and smooth spectral warping reduce error under most settings, albeit with different patterns of benefit. It is worth emphasizing that this paper defines the “robust performance interval” as a contiguous high-performance region with minor performance fluctuations within a neighborhood, rather than an incidental single-point extremum. Consequently, the discussion of augmentation parameters focuses on the range of stable benefits, rather than on the sharpest peak achieved in a single run.

The ablation results show that performance gains came from both data augmentation and ensemble integration. Relative to the non-augmented baseline, Gaussian-noise augmentation reduced average RMSE by 11.62%, 14.55%, and 17.14% for PLS, ridge regression, and XGBoost, respectively. Smooth spectral warping also improved model performance within an appropriate range, with average RMSE reductions of 10.61%, 12.85%, and 17.54% for the same three models. These results indicate that moderate perturbations helped the models learn more stable spectrum–chlorophyll relationships, whereas stronger perturbations led to partial performance loss in some settings.

The three learners did not respond to augmentation in the same way. PLS and ridge regression varied less across augmentation settings, indicating relatively stable but less flexible behavior. XGBoost achieved the best single-model performance in several settings, but its error also changed more with augmentation intensity. This pattern suggests that the linear models mainly contributed stability, whereas XGBoost contributed nonlinear fitting capacity.

Weighted ensembling provided an additional improvement beyond the best individual model. Under the best composite-augmentation setting, the ensemble improved R² from 0.6186 to 0.6392 and reduced RMSE from 9.1387 to 8.8883 relative to the best individual model, XGBoost. This corresponds to an RMSE reduction of 2.74% and an R² increase of 0.0206. Across the 16 composite-augmentation combinations, the ensemble achieved an average RMSE reduction of 3.71% and an average R² increase of 0.0308 relative to the best individual model in each group. These results indicate that weighted integration further improved prediction accuracy after augmentation had already reduced part of the generalization error.

The gain was not limited to a single isolated parameter setting. Good performance was maintained within a broader region around noise ≈ 0.002–0.003 and warp ≈ 0.01–0.025, indicating that the method benefited from a relatively stable operating range rather than from one accidental optimum.

**Figure 17:**
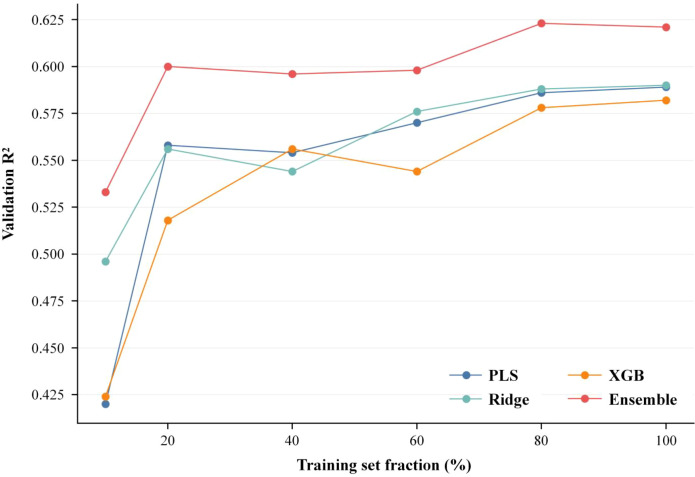
Sample-size response curves for the individual models and the weighted ensemble.

**Figure 17** demonstrates that linear models reach a plateau more quickly under smaller sample sizes, whereas XGBoost and the ensemble model continue to benefit from increasing sample size. This indicates that the ensemble method is not only effective at the current data scale but also possesses the potential for continued improvement with additional samples, thereby enhancing its scalability in practical applications.

**Figure 18:**
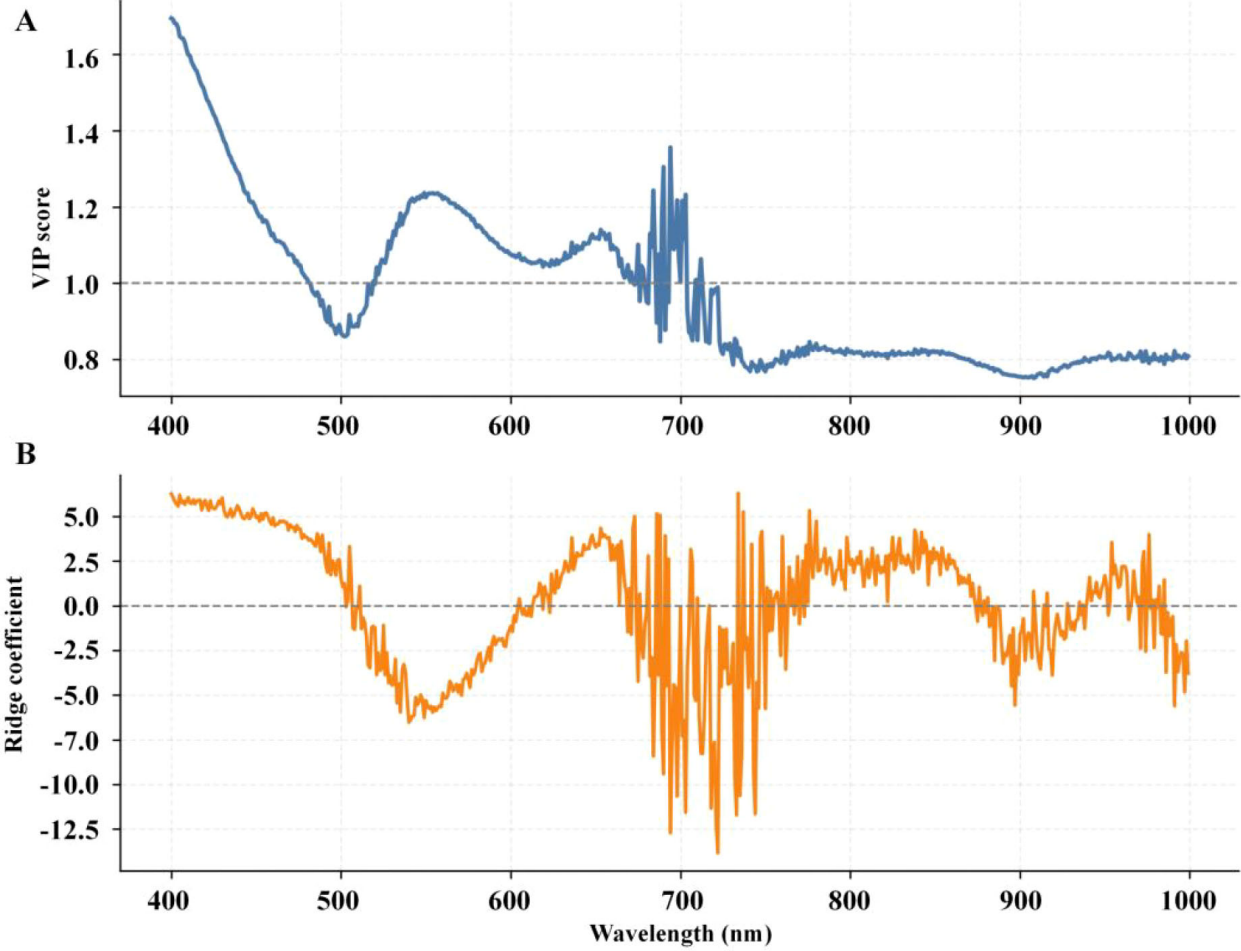
Wavelength information profiles for the best overall model setting. **(A)** PLS variable importance in projection scores. **(B)** Ridge regression coefficients.

**Figure 19:**
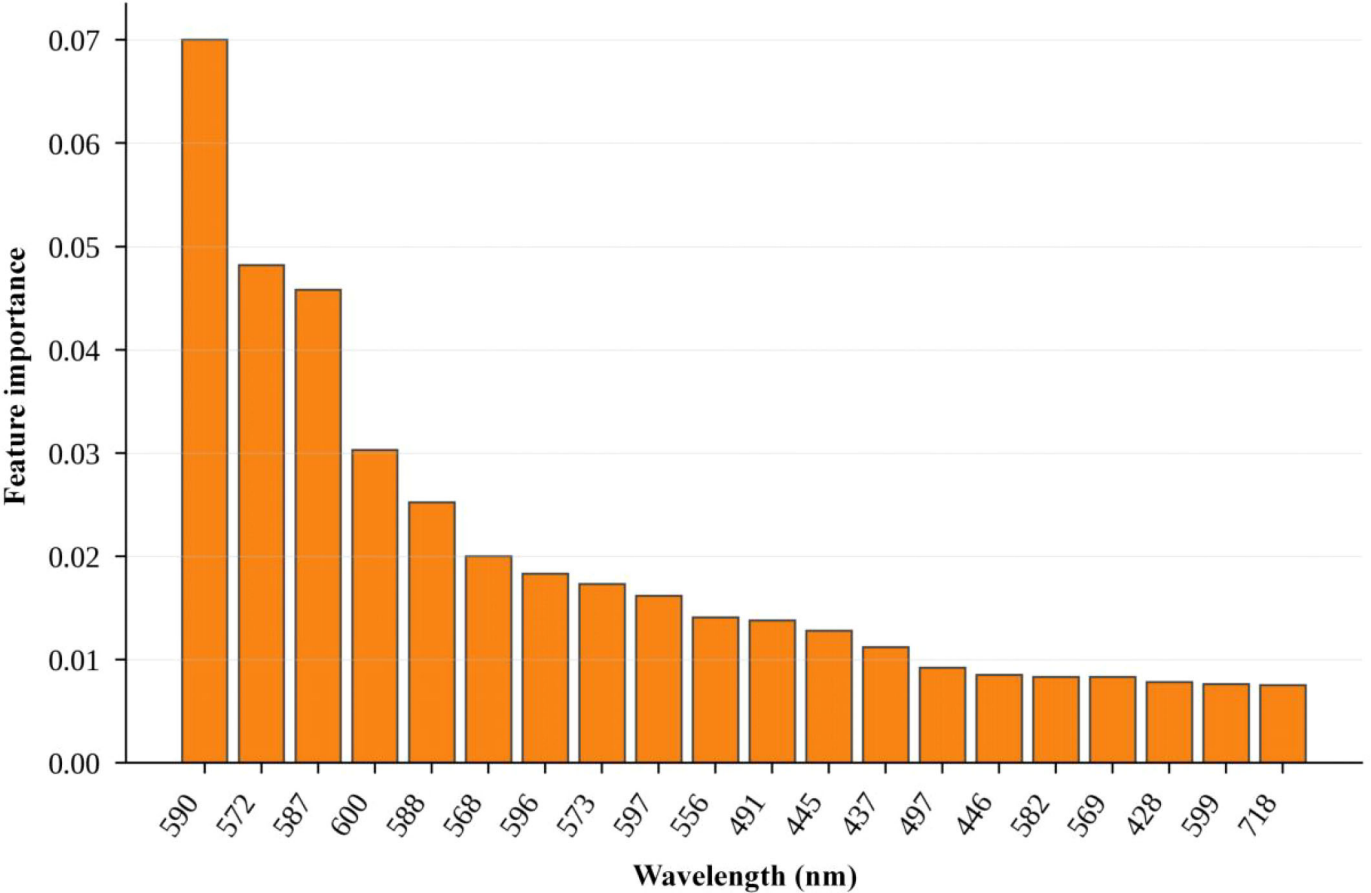
Top 20 XGBoost wavelength importances mapped to physical wavelengths.

**Figures 18** and **19** further show that the informative wavelengths were concentrated mainly in the visible-to-red-edge region. This distribution is consistent with known chlorophyll-sensitive spectral regions, suggesting that the performance gain was associated with more effective use of physiologically relevant spectral information rather than with arbitrary parameter tuning alone.

Overall, the results in this paper are consistent with the conclusions of Huang et al. (2023); Chen et al. (2024), and Guo et al. (2025) in terms of improvement logic, namely that effective hyperspectral chlorophyll estimation often requires simultaneously addressing both input distribution mismatch and model error complementarity. The difference lies in the fact that this study employs physically interpretable composite augmentation and heterogeneous weighted integration, rather than solely relying on deeper model structures or more complex feature engineering. Therefore, although this study cannot claim to outperform all previous work in absolute metrics, the present results indicate that physically constrained augmentation combined with heterogeneous weighted integration can provide a stable improvement pathway under the data and validation framework used here.

### 4.3 Limitations and future prospects

The external validation results are lower than those obtained on the internal validation set. Although composite augmentation and weighted ensemble learning improve model stability within the public dataset, cross-source prediction remains affected by sample distribution shifts, differences in sampling conditions, discrepancies in instrument responses, and incomplete consistency in reference value measurement chains. Therefore, subsequent work needs to expand sample sources, increase scenario coverage, and introduce stronger transfer constraints or source calibration strategies to further enhance the model’s external generalization capability and absolute comparability.

## 5 Conclusions

This paper does not aim for single-model optimization, but rather constructs a reproducible workflow for small-sample leaf chlorophyll hyperspectral estimation: first, unifying the matching relationship between sample identifiers and chlorophyll reference values; then applying physically constrained Gaussian noise and smooth spectral warping exclusively to the training set; followed by unified hyperparameter tuning and weighted ensemble of XGBoost, PLS, and Ridge. The revised manuscript further clarifies the comparability boundaries of the target variable and the interpretation of external validation. Results show that the proposed framework achieves R² = 0.6392 and RMSE = 8.8883 on the validation set, while maintaining a moderate positive correlation (R² = 0.498, RMSE = 9.801) on independent external data, indicating a certain degree of fidelity for cross-source samples. However, it also suggests that further improvements in external generalization and absolute comparability still require data from a wider range of sources, multiple locations, and diverse measurement chains.

## Data Availability

The raw data supporting the conclusions of this article will be made available by the authors, without undue reservation.
